# Development of Novel Zn^2+^ Loaded Nanoparticles Designed for Cell-Type Targeted Drug Release in CNS Neurons: In Vitro Evidences

**DOI:** 10.1371/journal.pone.0017851

**Published:** 2011-03-23

**Authors:** Andreas M. Grabrucker, Craig C. Garner, Tobias M. Boeckers, Lucia Bondioli, Barbara Ruozi, Flavio Forni, Maria Angela Vandelli, Giovanni Tosi

**Affiliations:** 1 Department of Psychiatry and Behavioral Sciences, Stanford School of Medicine, Stanford University, Stanford, California, United States of America; 2 Institute for Anatomy and Cell Biology, Ulm University, Ulm, Germany; 3 Te.Far.T.I. Group, Pharmaceutical Technology, Department of Pharmaceutical Science, University of Modena and Reggio Emilia, Modena and Reggio Emilia, Italy; The Mental Health Research Institute of Victoria, Australia

## Abstract

Intact synaptic function and plasticity are fundamental prerequisites to a healthy brain. Therefore, synaptic proteins are one of the major targets for drugs used as neuro-chemical therapeutics. Unfortunately, the majority of drugs is not able to cross the blood–brain barrier (BBB) and is therefore distributed within the CNS parenchyma. Here, we report the development of novel biodegradable Nanoparticles (NPs), made of poly-lactide-co-glycolide (PLGA) conjugated with glycopeptides that are able to cross the BBB and deliver for example Zn^2+^ ions. We also provide a thorough characterization of loaded and unloaded NPs for their stability, cellular uptake, release properties, toxicity, and impact on cell trafficking. Our data reveal that these NPs are biocompatible, and can be used to elevate intracellular levels of Zn^2+^. Importantly, by engineering the surface of NPs with antibodies against NCAM1 and CD44, we were able to selectively target neurons or glial cells, respectively. Our results indicate that these biodegradable NPs provide a potential new venue for the delivery Zn^2+^ to the CNS and thus a means to explore the influence of altered zinc levels linked to neuropsychological disorders such as depression.

## Introduction

The majority of drugs used as neuro-chemical therapeutics target synaptic proteins. Unfortunately, a high number of drugs are not able to cross through the blood–brain barrier (BBB) [1]. The transmissivity of this epithelial structure is restricted by the presence of tight junctions (TJ) that connect the cerebral endothelial and epithelial cells of the choroids plexus. Additionally, glial cells are found surrounding the surface of the capillaries, which cohere the endothelial cells, producing an electrical resistance much higher than that of other systemic endothelia [2]. Recent studies have demonstrated a non-invasive method of drug delivery to the CNS, based on the use of biodegradable Nanoparticles (NPs). Injectable nanoparticulate drug carriers made of poly-lactide-co-glycolide (PLGA), and specifically modified with ligands were shown to be able to cross the blood–brain barrier (BBB), thus representing an important potential tool for treatment of neurological diseases [3], [4].

In particular, this new strategy for NPs-brain targeting is based on the surface engineering of NPs, using a glycopeptides (g7)–derived PLGA [3], [5], [6]. The attachment of ligands for CNS targeting and/or fluorescent markers on the surface of NPs allows evaluating and influencing their properties both *in vitro* and *in vivo*. These engineered NPs (BBB-NPs) bear the possibility to deliver different kinds of drugs to the brain with a high rate of efficiency (13–15% of the injected dose) [7]. Thus, by encapsulation of drugs into BBB-NPs a significant improvement of brain delivery can be expected.

In recent years, there has been mounting evidence that suggests a correlation between zinc deficiency and clinical depression [8], [9]. Clinical studies as well as work with animal models indicate that zinc levels and some neuropsychological disorders are functionally linked. Zinc deficiency, for example, has been shown to induce depression- and anxiety-like behaviors, while zinc supplementation has been used to treat depression. Intriguingly, zinc administration improves the efficacy of antidepressant drugs in depressed patients [10] and the level of zinc at synapses in the hippocampus [11]. Thus, while zinc deficiency may have a critical role in the development of mood disorders and may serve as a putative model of depression in mice, zinc supplementation may be important in the treatment of these disorders [12].

Given the therapeutic potential of NPs, in the present study, we have investigated whether un-modified PLGA NPs (P-NPs) or BBB-NPs specifically targeted to neurons or glia cells could be used to encapsulate and deliver Zn^2+^ into cells. Our data demonstrate that this strategy is feasible creating a novel platform not only for understanding the role of zinc in neuronal function, but also the delivery of drugs into the CNS and ultimately the treatment of neurological diseases.

## Results

### NPs do not affect cell viability or neuronal morphogenesis

**Figure 1:**
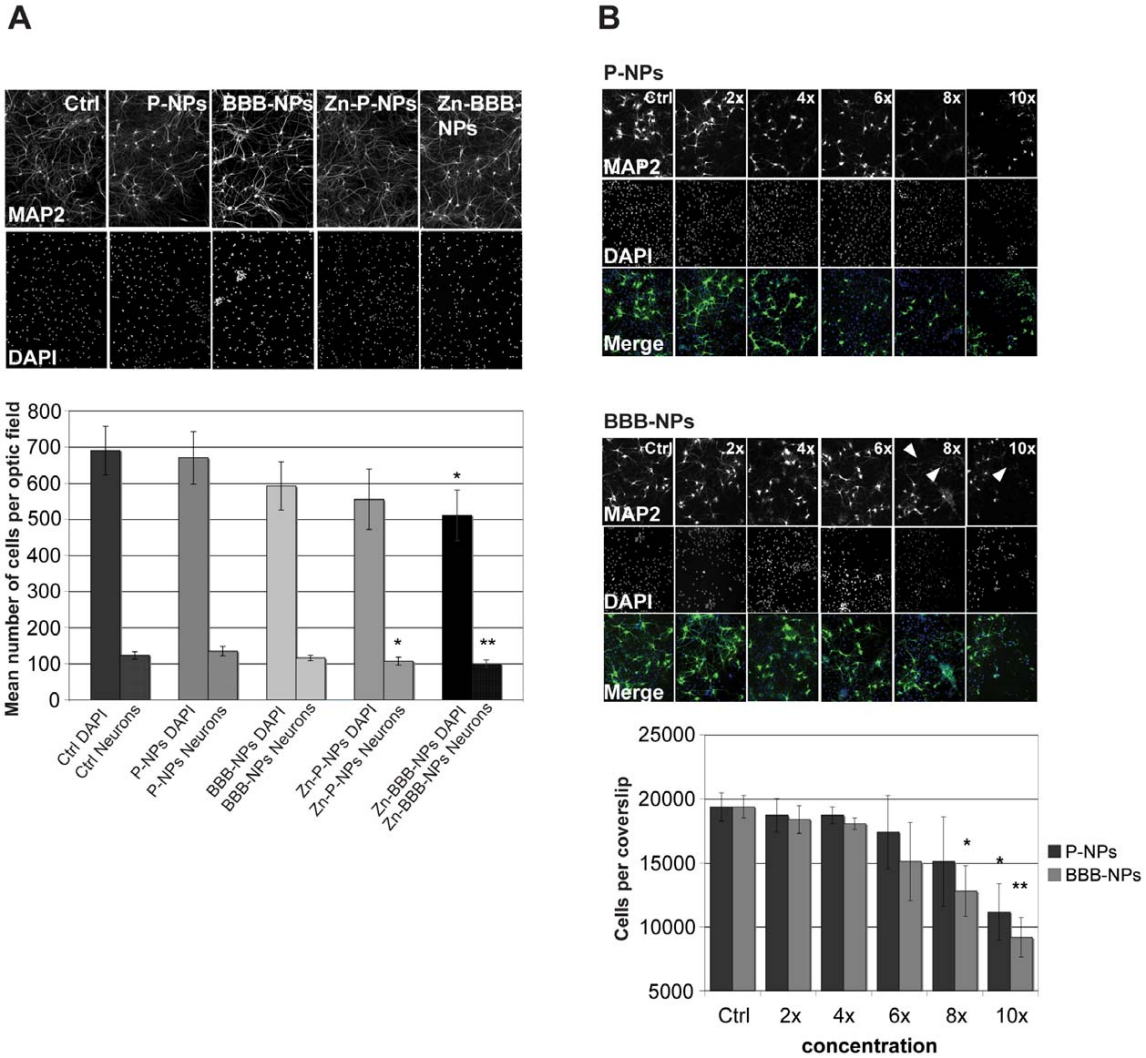
Cell viability after treatment with NPs. A) Mixed neuronal cultures containing glia cells and hippocampal neurons were plated and grown until DIV14. Cells were treated with the following NPs: “P-NPs” (empty NPs without ligand), “BBB-NPs” (un-loaded g7 ligand coated NPs), “Zn-P-NPs” (NPs encapsulating Zn^2+^) or “Zn-BBB-NPs” (g7 ligand coated NPs encapsulating Zn^2+^) (all: 625 µg NPs per ml), at DIV7. Upper panel: cells were stained with antibodies against the dendritic microtubule associated protein MAP2 and DAPI. The number of neurons (MAP2 positive cells) and the overall cell number (DAPI staining) per optic field was measured. Neither P-NPs nor BBB-NPs affected cell number as quantified in the lower panel. A small but significant reduction in cell number was observed in Zn-NPs (neurons) and Zn-BBB-NPs treated cells. B) Neuronal/glial cultures were plated at 20,000 cells per coverslip and treated for 7 days with different concentrations of P-NPs and BBB-NPs (e.g. multiples of 625 µg NPs per ml applied to control cells (Ctrl)). The cells were treated at 7DIV fixed at DIV14 and labeling for MAP2 and nuclei (DAPI) was performed. Lower panel: quantification of DAPI positive cells/optical field of view (40× mag). Only healthy nuclei were counted. At 10× 625 µg/ml (6.25 mg/ml), there is a significant reduction in cell number of cells treated with P-NPs. Cell-number reduction with BBB-NPs treated cells is seen at 8× 625 µg/ml (5 mg NP/ml). Arrows indicate neurons that underwent cell death.

Although in previous studies, NPs have been shown to cross the BBB, whether they are actively taken up by neurons and/or adversely affect cell viability is poorly investigated. To explore these possibilities, we examined the effect of adding NPs to dissociated neuronal/glial cultures. In an initial set of experiments designed to assess their affect on cell viability, cultures were treated with one of four preparations [unloaded P-NPs, unloaded BBB-NPs, Zn^2+^ loaded P-NPs (Zn-P-NPs) or Zn^2+^ loaded BBB-NPs (Zn-BBB-NPs)] starting at DIV7 (625 µg NPs per ml) (Fig. 1A) and compared to untreated control cells at DIV14.

Cells treated with unloaded P-NPs and BBB-NPs exhibited no difference in their morphology, e.g. dendrite branching measured by determining the “dendritic complexity index” DCI [13] or the number of synapses per unit length of dendrites (Fig. S1). The findings also allow to dismiss the possibility of a possible toxic effect of glycopeptides covering NPs surface (BBB-NPs) since there is no significant difference between viability-results with empty P-NPs and empty BBB-NPs. Furthermore, the number of neurons assessed by anti-MAP2 staining as well as overall cell number per field of view including glial cells assessed by DAPI staining was not changed compared to control cells. Cells treated with NPs containing Zn^2+^ (Zn-P-NPs and Zn-BBB-NPs) show a slight but significant reduction in glial and/or neuronal cell number following the addition of Zn-BBB-NPs or Zn-P-NPs, respectively. Since cells treated with empty P-NPs do not show this decrease in neurons, the effect is likely due to an increase in intracellular zinc levels and not by the treatment with NPs itself. The intracellular zinc concentration might have reached already toxic levels for neurons but not for glial cells (Zn-P-NPs) or both, neurons and glial cells (Zn-BBB-NPs) due to increased cellular uptake of Zn-BBB-NPs (Fig. 1A).

To further investigate the influence of NPs on cell viability, cells were plated with a density of 20,000 cells per coverslip and treated with different NP concentrations of “P-NPs” and “BBB-NPs”, i.e. Ctrl (625 µg NPs per ml), 2×, 4×, etc. compared to the concentration used in the first cell viability experiment (Fig. 1B). The cells were fixed at DIV14 and the number of cells was assessed. Beginning with the 6× concentration, the standard-deviation becomes larger, meaning that there were already some optic fields per/coverslip, where cell viability was reduced while in other regions cells remained healthy. However, at 8× (“BBB-NPs”) and 10× (“P-NPs” and “BBB-NPs”) concentrations of NPs, there is a significant reduction in cell number in all fields of view.

### Neurons and glial cells endocytose nanoparticles

**Figure 2:**
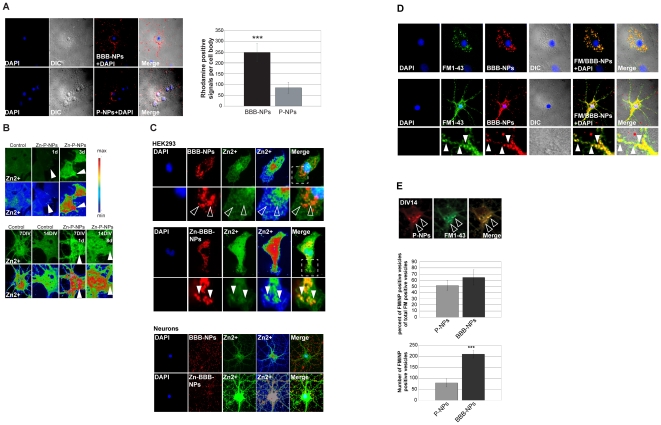
Subcellular localization of NPs in HEK293 cells and neurons. A) Images of hippocampal cultures treated with NPs (left panel). Nanoparticles without ligand (P-NPs) or coated with BBB-ligand (BBB-NPs) associate with cells. The distribution pattern is consistent with NPs both on the surface and within cells. Quantification of Rhodamine fluorescent signals per cells shows a ratio of BBB-NPs to P-NPs of 3.15 (n = 10) (right panel). B) Changes in the intracellular zinc levels of HEK293 (upper panel) and neuronal (lower panel) cells after addition Zn-P-NPs were detected by loading cells with Zinypr-1. Bright fluorescent vesicular structures were visible (arrows) in HEK293 cells as well as hippocampal neurons after application of Zn-P-NPs compared to untreated (control) cells. The bottom row of images in each panel shows heat maps revealing regional differences in zinc levels. C) Fluorescent images of HEK293 (upper panels) or neurons (lower panel) assessing the relative distribution of NPs and zinc. Zinypr-1 was used to detect Zn^2+^, and Rhodamine fluorescence to detect the distribution of Rhodamine labeled NPs. The zinc-signal colocalizes with fluorescent NPs in Zn-BBB-NPs treated cells (HEK293: middle panel, full arrows and Neurons: lower panel) but not in unloaded BBB-NPs (empty arrows, upper panel). D) Fluorescent images of glial cells (upper panel) and hippocampal neurons (lower panel) treated with Rhodamine-conjugated BBB-NPs and FM1-34FX. BBB-NPs fluorescence is observed colocalizing with FM1-43 FX within endocytotic vesicles (arrows). E) Fluorescent P-NPs can - similar to BBB-NPs - be found inside the cell soma co-localizing with FM 1-43 FX within endocytotic vesicles. Quantitative analysis of the percent of vesicles colabeled with NPs and FM dye in comparison to the total pool of FM-labeled vesicles reveals that no significant differences between P-NPs and BBB-NPs can be measured. However, the total number of FM/NP vesicles is significantly increased in BBB-NP treated cells vs. P-NP treated cells.

To explore the fate of NPs added to our neuronal/glial cultures, we took advantage of the fluorescent labeling of both unloaded P-NPs and BBB-NPs (by using tetra-methyl rhodamine conjugated PLGA in the formulation of the NPs). The results reveal that unloaded P-NPs and BBB-NPs readily associated with cultured cells (Fig. 2A). Interestingly, BBB-NPs were found associated to cells in higher amount compared to P-NPs (Fig 2A), perhaps due to the presence of the glycopeptides coating on the surface of BBB-NPs. To investigate whether cells take up NPs, we used Zn^2+^ loaded NPs and Zinpyr-1, a zinc staining fluorophore, to visualize intracellular zinc-ions. Interestingly, in contrast to untreated as well as ZnCl_2_ supplemented cells (data not shown), bright fluorescent vesicular structures were visible in HEK293 cells and hippocampal neurons (Fig. 2B, arrows and Fig. S2A) after supplementation of the growth medium with Zn^2+^ loaded Zn-P-NPs or Zn-BBB-NPs (data not shown). Monitoring the distribution of the rhodamine-conjugated PLGA, we observed a strong colocalization with Zinpyr-1 fluorescent puncta. These data are consistent with the internalization of NPs into Zn^2+^ rich intracellular organelles/vesicles (Fig. 2C). This conclusion is furthermore consistent with the selective co-localization of the zinc signal with fluorescently tagged Zn-BBB-NPs treated cells (Fig. 2C, full arrows) but not cells treated with unloaded BBB-NPs (Fig. 2C, empty arrows).

In order to study the mechanism of entrance and the localization of BBB-NPs within cells, we used fluorescent BBB-NPs together with FM1-43FX dye (Fig. 2D). FM1-43FX is a fixable membrane probe widely used for monitoring recycling vesicles [14]. In these experiments, fluorescently labeled BBB-NPs (Fig. 2D) and P-NPs (Fig. 2E) were found to decorate the same vesicular structures labeled with FM1-43FX consistent with the model of NPs entering cells by endocytosis (Fig. 2A,D,E). Analysis of the amount of vesicles harboring NPs compared to the total pool of labeled vesicles shows that no differences between P-NPs and BBB-NPs can be seen. However, the total number of FM1-43FX+NP positive vesicles was significantly increased in BBB-NP treated cells (Fig. 2E). These findings suggest that the PLGA coating might enhance endocytosis of NPs. Additionally, we monitored the fluorescent pattern of BBB-NPs treated cells over a time-course of 7 d. This revealed that the initial punctate pattern becomes more diffuse over time, consistent with the slow degradation of BBB-NPs resulting in cells with higher background fluorescence (Fig. S2B).

Taken together, these data indicate that BBB-NPs have the capacity to release their content intracellularly.

### Characterization of Zn^2+^ loaded NPs

As a biodegradable polymer, NPs are predicted to slowly release their content over time. We were thus keen to characterize the rate of zinc release both *in vitro* and *in vivo*. For all studies outlined below, batches of NPs were characterized for their size, surface charge and general shape by means of Photon Correlation Spectroscopy (PCS) methods and SEM analysis. All NPs (independently from the surface modifiers or the loading) show an average size of 190–210 nm, with overall surface charge (expressed as zeta-potential, z-p) close to neutrality (z-p ranging from −0.5 to −10 mV). This value was maintained also after re-suspension of all kinds of lyophilized NPs. Moreover, a study on the content and release in 7.4 pH buffer was performed on Zn^2+^-loaded P-NPs (Zn-P-NPs) and BBB-NPs (Zn-BBB-NPs) evidencing a mean of 2.6 mg of Zn^2+^ (detected both by colorimetric and atomic absorbance technologies) per 100 mg of NPs. Moreover, surface analysis (i.e. ESCA analysis) of the antibody-engineered NPs (Anti-NCAM1-NPs and Anti-CD44-NPs) demonstrated the efficacy of the surface modification process.

### Characterization of the *in vitro* release of Zinc from NPs

ZnSO_4_. 7H_2_O was encapsulated in both P-NPs and BBB-NPs (mean size close to 200 nm, PDI of 0.204 and a z-p of −6.13 mV) with a final content of 2.6 mg of Zn^2+^-ions per 100 mg of NPs. Both NP samples showed similar features (with respect to loading and chemico-physical properties). To assess the timescale, in which Zn-P-NPs and Zn-BBB-NPs release Zn^2+^ *in vitro*, both types of Zn-loaded NPs were suspended in Neurobasal Medium +Glutamine +B27 (NB++). A 1.5 µM basic Zn^2+^ concentration of NB++ was measured using plasma mass-spectrometry.

**Figure 3:**
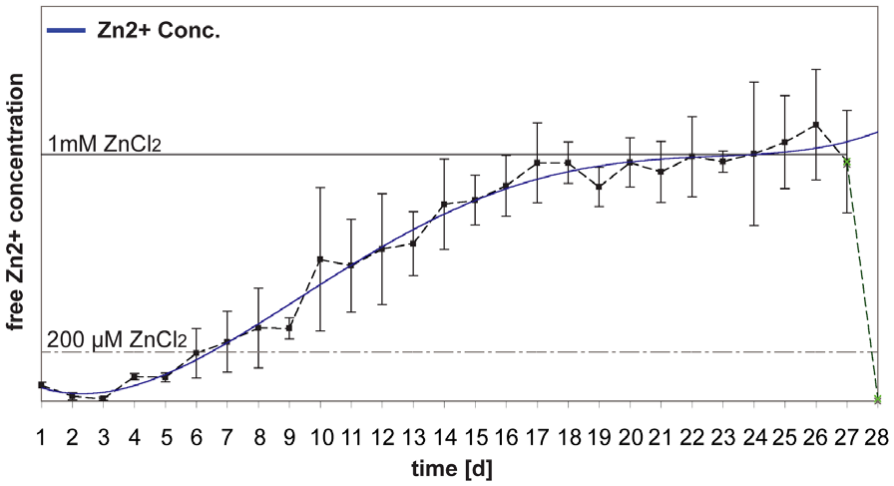
NPs release Zn^2+^ over time. Quantification of the rate of zinc released from NPs in vitro by monitoring free zinc levels with Zinypr-1 fluorescence. The blue line indicates the trend-line for the obtained data-points. The green line shows an overall drop of fluorescence after treatment with TPEN that competitively binds free zinc with higher affinity. The fluorescence intensity of Zinpyr-1 was measured every 24 hrs and correlated to the fluorescence of medium with known zinc concentration.

A suspension with an amount of Zn-loaded NPs (Zn-P-NPs and Zn-BBB-NPs) approx. encapsulating 1 mM Zn^2+^ was prepared, added to the medium and incubated for 28 days at 37°C. Each day, the zinc concentration was measured using Zinpyr-1, (C_46_H_36_Cl_2_N_6_O_5_) a fluorescent sensor for Zn^2+^ with a high specificity and affinity for Zn^2+^ (K_d_ = 0.7±0.1 nM) (Fig. 3). Since Zinpyr-1 is not able to enter or to be adsorbed by NPs, the rise in fluorescence is due to NP-Zn^2+^ release, a value that correlates directly. After 28 days, TPEN (N,N,N′,N′-Tetrakis(2-pyridylmethyl)ethylenediamine) was added to the samples. TPEN is a water-soluble and cell membrane permeable zinc chelator with very high affinity for Zn^2+^ (K_d_ for Zn^2+^ binding to TPEN is 6.3 * 10^−16^ M at pH 7.6). A drop in fluorescence shows that the fluorescence of Zinpyr-1 indeed was due to the presence of Zn^2+^ in the medium. Since Zinpyr-1 only fluoresces upon binding Zn^2+^, the loss of Zn^2+^ due to chelation causes this drop in fluorescence.

The results of the release of Zn^2+^ suggest that Zn-P-NPs and Zn-BBB-NPs degrade over time, producing a sustained release: in particular, during the first 16 days, a slightly faster release of Zn^2+^ is visible compared to day 16–22, while, after 22 days, nearly all Zn^2+^ is released (Fig. 3). The release profile could be due to a degradation/diffusion process happening on NPs architecture. Since the release of Zn^2+^ from both types of NPs is almost the same, we chose to show only the release of Zn-P-NPs, in order to avoid overlapping and redundancy of results.

### Release of Zinc from NP in cultured cells

To assess whether an intracellular environment alters the time release properties of NPs, we monitored changes in intracellular Zn^2+^ in HEK293 cells and Hippocampal neurons treated with NPs loaded with Zn^2+^ (Zn-P-NPs and Zn-BBB-NPs). As above, the Zinpyr-1 fluorescent probe was loaded into cells by its addition to the culture medium. Zinpyr-1 is able to penetrate cell membranes.

**Figure 4:**
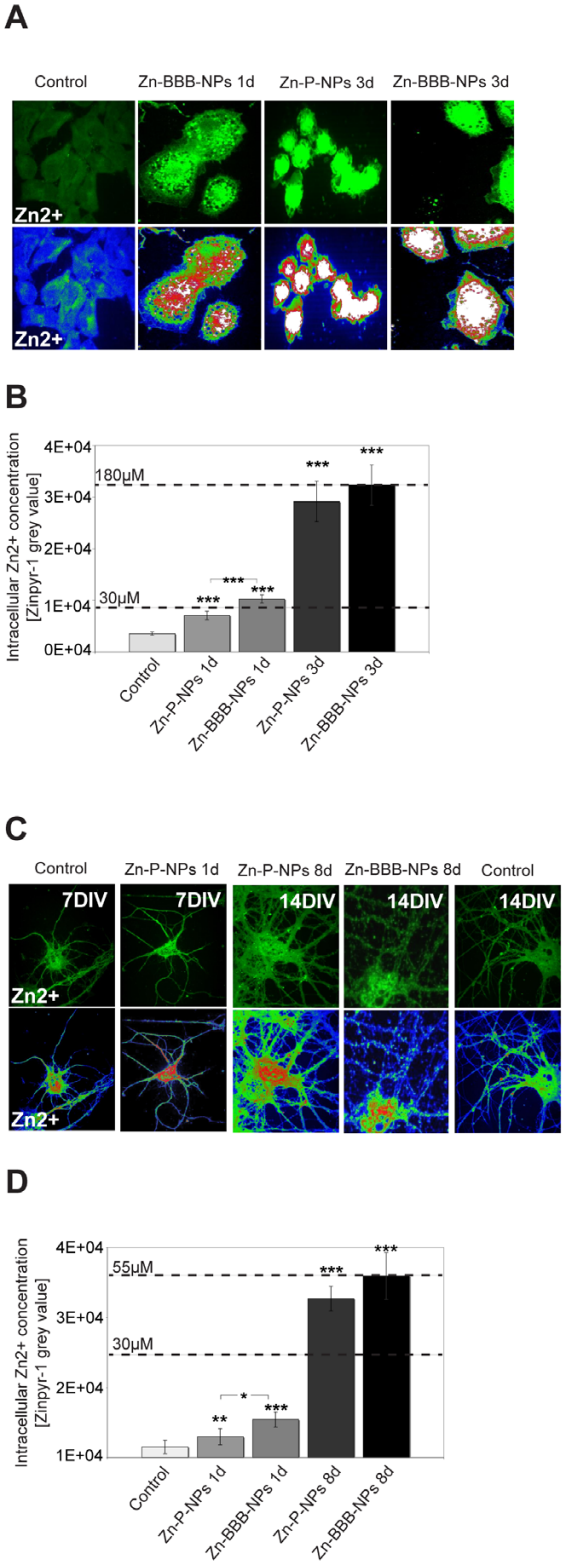
Intracellular Zn^2+^ increase over time. A) Changes in intracellular zinc levels of HEK293 cells incubated with Zn-P-NPs or Zn-BBB-NPs for 1–3 days (End concentration: 1 mM after complete degradation) monitored by Zinypr-1 fluorescence (upper row). Color-coding of Zinpyr-1 fluorescence (lower row) reveals changes in the intracellular zinc concentration. B) Quantification intracellular zinc with Zinypr-1 (for control and ZnCl_2_ reference see Fig S4.). Zinc levels in cells treated with Zn-BBB-NPs (41 µM) are significantly higher after one day compared to cells treated with Zn-P-NPs (22 µM). After 3 days, a final zinc concentration of 160 µM (Zn-P-NPs) and 180 µM (Zn-BBB-NPs) was reached. C) Hippocampal neurons were incubated with Zn-P-NPs and Zn-BBB-NPs at 6DIV and zinc levels determined by monitoring changes in Zinpyr-1 fluorescence (upper row and lower row heat-maps) at 7DIV and 14DIV (End concentration: 250 µM after complete degradation). D) After one day, Zn-P-NPs (7 µM) show a significantly lower level of intracellular zinc compared to Zn-BBB-NPs treated cells (10 µM). After 8 days, a final zinc concentration of 48 µM (Zn-P-NPs) and 55 µM (Zn-BBB-NPs) was reached.

In initial experiments, we examined the rate of intracellular NPs-Zn^2+^ release in HEK293 cells, a wildly used fibroblast cell line that shares many properties of immature neurons [11]. Here, HEK293 cells were incubated with a 30 µM ZnCl_2_ solution and a suspension of Zn-P-NPs and Zn-BBB-NPs (End concentration: 1 mM Zn^2+^ after NP degradation in DMEM) (Fig. 4A,B). A final zinc concentration was calculated using grey values of Zinpyr-1 fluorescence correlating to the local zinc concentration. The background fluorescence of untreated HEK293 cells was subtracted and cells treated only with 30 µM ZnCl_2_ used as reference (Fig. S4). After 3 days, a final zinc concentration of 180 µM released by Zn-BBB-NPs was reached (Fig. 4A,B). Compared to the expected Zn^2+^ release-curve (Fig. 3), this concentration should have been reached only after 6–7 days. Thus, compared to *in vitro* release of Zn^2+^ from NPs, the release appears to occur faster in cells. This might be due to a faster degradation of intracellular NPs. In line with this, Zn-BBB-NPs that show increased cellular uptake compared to Zn-P-NPs also lead to a significantly higher increase of the intracellular zinc concentration after 1 d. Not unexpectedly, after 3 days, HEK293 cells showed signs of distress and underwent cell death. This is consistent with published literature showing that an intracellular free zinc concentration of this magnitude is cyto-toxic [15], [16] (and Fig S3).

### Zinc release in rat hippocampal neurons

In a parallel set of cell-based experiments, we investigated the extent of Zn^2+^ release from NPs in Hippocampal neurons. As above, cells were incubated with a 30 µM ZnCl_2_ solution as zinc-loading control (Fig. S4) and a suspension of Zn^2+^ loaded NPs (Zn-P-NPs and Zn-BBB-NPs) (End concentration: 250 µM Zn^2+^ after NP degradation in Neurobasal +B27, +Glut) (Fig. 4C,D). To maintain cell viability and ensure the growth and maturation of primary hippocampal culture cells for 14DIV, the amount of NPs loaded was reduced compared to the solution taken for HEK293 cells. Although the local Zn^2+^ concentration at synapses after synaptic activity can reach 300 µM, prolonged exposure to high free Zn^2+^ concentration causes cell death [15]–[17]. This is nicely illustrated in supplemental data where we find that neurons are more sensitive to free Zn^2+^ compared to HEK293 cells, however concentrations higher than 160 µM lead to cell death in both cell cultures (Fig. S3).

In these experiments, the background and 30 µM ZnCl_2_ fluorescence was measured at 7DIV and 14DIV and since no difference was obtained (Fig. S4), the average is used for the quantification of intracellular Zn^2+^ elevation (Fig. 4C,D). After application of Zn^2+^ loaded NPs, neurons display an increase in intracellular zinc concentration. The zinc level can be reduced by replacing the growth medium with medium without NPs (data not shown). This leads to Zn^2+^ efflux due to lower extracellular Zn^2+^ concentrations in the culture medium (0.093 µg Zn^2+^/ml = 1.42 µM for Neurobasal medium with addition of B27, Pen/Strep and Glutamine measured by Plasma-Massspectrometry). As in HEK293 cells, Zn-BBB-NPs in neuron elicits a higher intracellular level of zinc compared to Zn-P-NPs treated cells (Fig. 4D).

### Targeting Nanoparticles to specific neural cell populations

**Figure 5:**
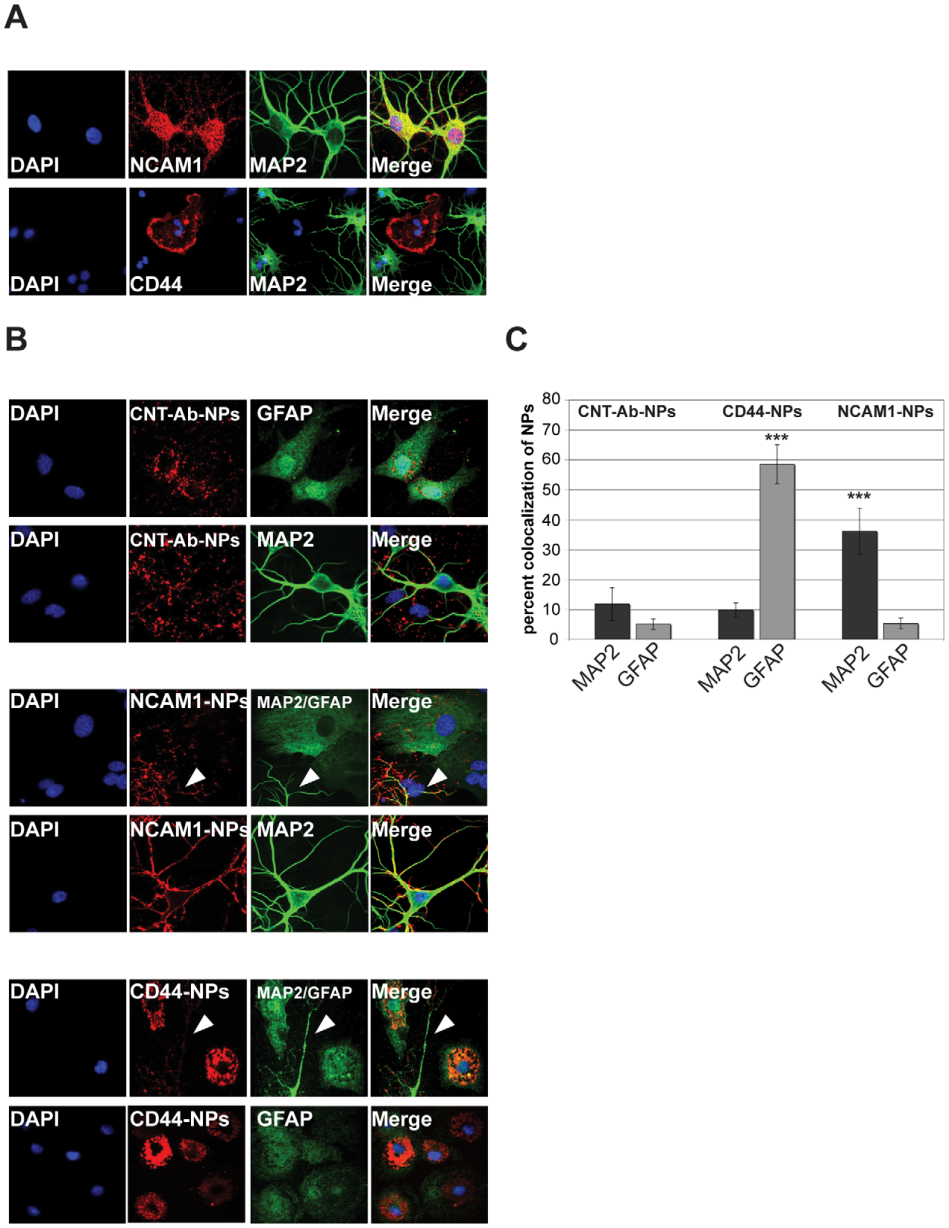
Cell type specific targeting of BBB-NPs. A) Immuncytochemistry of hippocampal neurons 14DIV stained with antibodies against NCAM1, CD44 and MAP2. The neuronal protein NCAM1 is found to label neurons that are also immuno-positive for MAP2 (upper row of images). CD44 positive glia are not labeled with MAP2 (lower panel). B) Fluorescent images of neuronal/glial cultures treated with TMR-labeled NPs (red) coated with antibodies against NCAM1 or CD44. Antibodies against MAP2 and the glial specific intermediate filament associated protein GFAP (green) were used to label neurons and glia in these cultures. Unmodified NPs (CNT-Ab-NPs) association with glial cells as well as neurons (upper panel). NCAM1-NPs preferentially label MAP2 positive neurons (arrow, middle panel) but not GFAP positive glia. In contrast, CD44-NPs preferentially label glial cells and a reduced labeling of neurons (arrow, lower panel) is seen. Note that the anti-CD44 ligand appears to promote NPs endocytosis. C) Quantification of percent colocalization (yellow) between NPs (red) and MAP2 (green) or GFAP (green) (n = 10). CD44-NPs show significantly higher colocalization with GFAP compared to NCAM1-NPs and CNT-AB-NPs. In contrast, NCAM1-NPs show significantly higher colocalization with MAP2 compared to GFAP and CNT-Ab-NPs. CD44-NPs and NCAM1-NPs show similar colocalization values like CNT-Ab-NPs for MAP2 (CD44-NPs) and GFAP (NCAM1-NPs).

Based on these experiments, it can be assumed that the endocytosis of un-modified NPs (P-NPs and BBB-NPs) in the CNS will occur in neurons as well as glial cells. However, a more targeted drug release may be desired. Given that P-NPs can be modified with almost any combination of ligands, we explored the possibility of achieving cell type specific targeting. As a proof principle experiment, we coupled NCAM1 and CD44 antibodies to the surface of NPs that recognize antigens on the surface of neurons and glia, respectively. As a control for antibody specificity, neuronal cultures were immuno-stained with antibodies against NCAM1, CD44 and MAP2 a neuronal specific microtubule associated protein. As expected, NCAM1 antibodies immuno-labeled MAP2 positive cells, whereas CD44 antibodies labeled glia cells (Fig. 5A).

We then applied fluorescent NPs conjugated with anti-NCAM1 (anti-NCAM1-NPs) or anti-CD44 antibodies (anti-CD44-NPs) to primary mixed hippocampal cultures to evaluate their targeting behavior in presence of both, glia and neuronal cells (Fig. 5B). As expected, control NPs without further modification (CNT-Ab-NPs) associated with glial cells and neurons to a similar degree (Fig. 5C). However, anti-NCAM1-NPs (NCAM-NPs) show reduced targeting to glial cells together with highly increased targeting to neurons stained for MAP2 (arrow Fig. 5B middle panel and C). In contrast, anti-CD44-NPs (CD44-NPs) show increased targeting to glial cells stained for Glial fibrillary acidic protein (GFAP) together with decreased targeting to neurons (arrow Fig. 5B lower panel and C). Interestingly, the anti-CD44 ligand not only increases targeting to glial cells, but appears to promote the endocytosis of NPs.

## Discussion

Non-invasive CNS drug delivery systems have been actively studied, especially with the development of colloidal carriers such as nanoparticles (NP) and liposomes. Indeed, reports in the literature show that these carriers if properly engineered, with a diameter around 100–200 nm, are able to cross the BBB without apparent damage [18], and can deliver drugs or genetic material into the brain [19], [20]. However, a selective biodistribution within the CNS is highly needed and this goal is far from being achieved. Nowadays, the use of a polymeric NP is one of the most promising approaches for CNS drug delivery [18], [21], [22], because polymeric NPs possess advantages with respect to free drug molecules or pro-drugs, such as a high drug-loading capacity [22]. In addition, NPs protect the embedded drugs against chemical or enzymatic degradation, thus increasing the chance for the active molecule to reach the target site. Only few polymers currently guarantee the safety of the polymer-based nanocarriers. Polylactide-co-glycolide (PLGA) or polylactide (PLA) polymers are biodegradable, biocompatible, FDA-approved and are therefore two of the most promising polymers for the preparation of NPs [18]. In this paper, we evaluated whether PLGA-based NPs could efficiently deliver Zn^2+^ to neuronal cells.

In brain chelatable Zn^2+^ has been detected in presynaptic vesicles of glutamatergic terminals and Zn^2+^ ions are released from the presynaptic site. At the postsynaptic site, Zn^2+^ ions are recruited into large macromolecular platforms within the PSD, assembled by scaffolding molecules such as ProSAP2/Shank3 and ProSAP1/Shank2, thus modulating the structure of the protein meshwork underneath the postsynaptic membrane [23]–[26]. In animal models, zinc exhibits antidepressant-like effects in the forced swim test, both in mice and rats and in tail suspension test [27]–[31], which are used for evaluation of antidepressant activity. Moreover, very low doses of zinc administered together with low, ineffective doses of imipramine or citalopram enhanced the antidepressant-like effect in this test [27], [32]. In humans, involvement of zinc in antidepressant therapy has some clinical correlates. It was shown that human depression is likely to be accompanied by lower serum zinc concentrations [33]–[35]. Subjects suffering from major depression showed significantly lower serum zinc levels than non-depressed controls, whereas patients with minor depression showed intermediate zinc levels [36]. Furthermore, a relationship between severity of depressive symptoms and decreased serum zinc concentration in postpartum depression was demonstrated [37] and zinc supplementation showed a significant reduction in anger-hostility and depression-dejection score in the Profile of Moods State (POMS) of women, suggesting that zinc supplementation may be effective in reducing anger and depression [38], [39].

Here, we show that PLGA-based NPs can be considered as a promising drug delivery system for the specific application of Zn^2+^ to CNS neurons. Several kinds of Zn^2+^ loaded or unloaded NPs (un-modified or modified for BBB crossing) were tested both in cultured fibroblast and primary neurons. The experiments show that NPs themselves are not toxic to cells, even at concentrations higher than those used for delivering Zn^2+^ (Fig. 1). In fact, cell viability is not affected below approx. 5000 µg/ml of NPs, a concentration much higher than that needed to efficiently deliver Zn^2+^ to cells. NPs decorated with the BBB-crossing ligand (BBB-NPs), a glycopeptide consisting of 7 aminoacids are slightly more toxic, however not at concentrations capable of dramatically increasing intracellular Zn^2+^ levels. Nevertheless, the data obtained should provide an important base line with respect to the evaluation of toxicity of unloaded NPs for future experiments.

Regarding NP mediated therapies, an important question is how, when and where NPs release their content, e.g. interstitially or perhaps following cellular uptake. In the event of endocytosis-mediated NP uptake, it is predicted that the forward trafficking of these endocytic vesicles to lysosomes could enhance release rates of zinc due to the low pH in lysosomes (Fig. 4). To test this hypothesis, we assessed the fate of Rhodamine labeled P-NPs and BBB-NPs after application to cells together with FM-43 labeling (Fig. 2D). Our results show that NPs associate with the surface of cells and then seem to appear in an endocytic compartment together with FM-1-43. This latter data indicates that they were endocytosed by these cells (Fig. 2D,E). Interestingly, it seems that the presence of BBB-ligand (glycopeptides) on the surface of BBB-NPs enhances the number of endocytotic events compared to un-modified NPs. The glycopeptides covering the surface of BBB-NPs exhibit a helix-like conformation [40] postulated to contribute to the entrance of BBB into cells by stimulating membrane curvature and thus endocytosis. To date, no data on the effect of this “membrane curvature effect” are available for neurons, but a membrane “hopping” type mechanism is likely [41]. Due to the fact that NPs are polymeric and not lipid based as liposomes, it is unlikely that they are able to fuse with cell lipid membranes. However, NPs stay in very close contact with the cell surface and released zinc could enter cells via specific or unspecific transporters/ion channel systems. While feasible, the rise in cell soma fluorescence associated with both Rhodamine-labeled BBB-NPs and P-NPs after 7 d, indicates that at least some of these NPs entered and released their content inside cells.

This conclusion is consistent with our results showing that BBB-NPs can be used to elevate the intracellular Zn^2+^ concentration in a cell-based assay (Fig. 4). Given that the release rate of encapsulated Zn^2+^ in NPs (Zn-NPs) measured *in vitro* (Fig. 3) showed that after approximately 3 weeks, nearly all NPs released their content, the elevation in zinc concentration might be due to an extracellular rise in Zn^2+^ or endocytosed NPs releasing Zn^2+^ inside the cell. However, in our cell-based experiments the release-rate of zinc is faster, and perhaps cell autonomous, due to their uptake. This is exemplified by the observed difference between BBB-NP and P-NP, where BBB-NPs are more readily taken up by cells, resulting in higher intracellular levels of zinc.

Since glial cells are known to clear the extracellular fluid of substances within the brain, having NPs that selectively target neurons or glial cells will be helpful in many ways. The extent to which glial cells participate in psychiatric disorders is currently only beginning to be explored, but a crucial role of glia in a wide range of neurological disorders has long been recognized. Although neurodegenerative disorders such as Parkinson's disease, Alzheimer's disease, ALS and Huntington's are caused by the death of neurons, glial cells have both a positive and negative influence on the progression of these diseases [42]–[45]. To differentially target neurons and glial cells, we designed antibody-engineered labeled NPs using antibodies directed against an extracellular epitope specific for neurons (NCAM1) and glial cells (CD44). These modified NPs demonstrate that a more selective targeting strategy can be used to enrich zinc or other drugs in a cell type specific manner. Intriguingly, in case of CD44, this ligand may influence endocytosis positively. Based on the results of this study, the targeting behavior, drug release properties and metabolism of the generated antibody-conjugated NPs can be investigated in an *in vivo* model as a next step for future application of NPs.

### Conclusions

In this study we have characterized a novel nanoparticle technology, based on PLGA NPs modified with glycopeptides known to promote the ability of NPs to cross the BBB *in vivo*. We demonstrated that both un-modified and BBB-crossing modified NPs, harboring substances released over time, are able to influence neuronal cells in culture. We could show that these NPs can efficiently deliver Zinc to cells at non-toxic concentrations. We also provided evidence that NPs can be easily modified so that they preferentially target specific cell populations, e.g. neurons versus glial cells, after crossing the BBB. This strategy should improve the delivery of drugs to the CNS. Thus, this work provides important base line data for future *in vivo* application of Zn^2+^ delivery by using modified biodegradable NPs.

## Materials and Methods

### Materials

ZnCl_2_, the Zn^2+^ chelator TPEN (*N,N,N′,N′*-tetrakis(2-pyridylmethyl)ethylenediamine) and Zinpyr-1 were purchased from Sigma-Aldrich. Primary antibodies were purchased from Chemicon (MAP2), Abcam (GFAP), Fisher Scientific (anti-CD44, clone OX-50), Lifespan Biosciences (NCAM1 extracellular domain) and Synaptic Systems (Homer1). Secondary antibodies Alexa488, 568 and 647 were from Invitrogen. Poly(D,L-lactide-co-glycolide) (PLGA RG502H) was used as received from the manufacturer (Boehringer-Ingelheim, Ingelheim am Rhein, Germany). BBB ligand (glycopeptides) was provided by EZ-Biolab, (Carmel, USA). PLGA conjugated with tetramethylrhodamine (TMR-PLGA) and conjugated with glycopeptides for BBB crossing (BBB-PLGA) was prepared as previously described [3], [6]. FM1-43FX dye was purchased from Invitrogen. A MilliQ water system (Millipore, Bedford, MA, USA), supplied with distilled water, provided high-purity water (18 MΩ). Unless otherwise indicated, all other chemicals were obtained from Sigma-Aldrich and were of analytical grade.

### Preparation of Nanoparticles

NPs were prepared as described in literature [46] with some modifications in the preparation procedure. For a typical formulation, 225 mg of ZnSO_4_ were dissolved in 0.25 ml of distilled water and emulsified in 2.5 ml of CH_2_Cl_2_ (containing 50 mg of a mixture of PLGA 503 H and BBB-PLGA (80∶20 w/w) (with or without 5–10 mg TMR-PLGA) by sonication over an ice bath using a probe sonicator (Misonix, MicrosonTM Ultrasonic Cell Disruptor XL, Opto-lab, Concordia, Mo, Italy) at 60 W output for 45 sec. The resulting primary emulsion was added to 5 ml distilled water containing PVA 1% (15000 MW, Sigma-Aldrich) and was sonicated for 60 sec at 100 W amplitude over an ice bath to form the double emulsion. Organic solvent was removed by stirring at room temperature for at least 1 h and finally purified by Hi-Speed Refrigerated Centrifugation (Beckman J21) at 14000 rpm for 10 min. Alternatively, we also obtained unloaded NPs (P-NPs and BBB-NPs) by omission of drugs within the inner aqueous phase. With this procedure we were able to obtain Zn-P-NPs (when using un-modified PLGA) and Zn-BBB-NPs (when BBB-PLGA was used). In conclusion, labeled (TMR-conjugation) BBB-NPs as well as NPs were obtained and purified to be tested in cell cultures.

Antibody engineered NPs were prepared starting from PLGA NPs, obtained as described before for P-NPs, and applying well known methodologies for Ab-surface engineering of NPs [47]. Briefly, in the presence of EDC (1-Ethyl-3-(3-dimethylaminopropyl)-carbodiimide, EDC) (170 mg) and N–Hydroxy-succinimide (NHS, 30 mg) to conjugate the free primary amine groups on the NPs surface with the carboxylic groups on the antibody molecules, desired amount of TMR-labeled P-NPs (50 mg) was suspended and stirred at RT for 1.30 hr in MES (2-(N-morpholino)ethanesulfonic acid, Sigma Aldrich) buffer with designated volume of Anti-NCAM1 Ab (50 µl of a 1 mg/mL stock solution) or Anti-CD44 Ab (50 µl of a 0.5 mg/mL stock solution) stock solution or a corresponding amount of buffer solution, in order to obtain respectively Anti-NCAM1-NPs, Anti-CD44 NPs and control group (CNT-Ab-NPs). After the reaction, the suspension of the engineered NPs were collected by centrifugation and further washed twice by distilled water for purification. Some amount of engineered NPs was re-suspended in ultrapure water for further characterization, while other NPs were lyophilized to prepare stocks (trealose was added as cryoprotector).

### NPs characterization

All the batches of NPs were characterized in their surface properties, size and shape. All the data collected (data not shown) demonstrated that the surface engineering (with Ab) of NPs or the loading with Zn did not affect the morphological features of the NPs, all having similar dimensions and shape. A scanning electron microscope (SEM) (XL-40 Philips, Eindhoven, The Netherlands) (×10,000) was used to evaluate the morphology of NPs. Before the SEM analysis, the samples were coated under argon atmosphere with a 10 nm layer of palladium gold (Emitech K550 Supper Coated, Emitech LTD, U.K.). NPs in distilled water were analyzed for particle size and zeta potential (z-p) by photon correlation spectroscopy and laser Doppler anemometry using a Zetasizer Nano ZS (Malvern, UK; Laser 4 mW He–Ne, 633 nm, Laser attenuator Automatic, transmission 100% to 0.0003%, Detector Avalanche photodiode, Q.E.>50% at 633 nm, T = 25°C). The results were normalized with respect to a polystyrene standard solution.

As scientifically established in literature, [47] the surface engineering of NPs (Anti-NCAM1 or Anti-CD44 NPs) was demonstrated by Electron Spectroscopy for Chemical Analysis (ESCA) study showing the presence of atoms (N) present on the surface of antibody-engineered NPs and thus proof of success of the surface engineering procedure. ESCA was performed on a 04-153 X-ray source analysis system (PHI, Uvalca-PHI, Tokyo, Japan) and an EA11 hemispherical electron analyzer (Leybold Optics, Germany), using MgKα1,2 radiations. The spectra were recorded in fixed retardation ratio (FAT) mode with 190 eV pass energy. The pressure in the sample analysis chamber was ca. 10−9 mbar. The data were acquired and processed using the RBD AugerScan 2. 1H-HRMAS NMR spectra were recorded on a Bruker Avance 400 instrument; D2O was added to the sample, which was then spun at 4000 Hz. All experiments were recorded at RT.

### Determination of NPs content

10 mg of NPs (both batches of BBB and plain NPs) loaded with ZnSO_4_ were dissolved in 1 ml of DCM and 5 ml of milliQ water were added to the organic solution. The organic solvent was removed by stirring at room temperature for at least 3 h and finally the aqueous solution was filtered through a syringe filter to eliminate the polymer insoluble in water. The final volume of the aqueous solution was adjusted to 25 ml with distilled water and 1 ml of this solution was diluted to 25 ml. The final aqueous solution was analyzed through atomic absorption spectrophotometry. A 5 mM Zn^2+^ containing stock solution (12.5 mg NPs/ml) was prepared. The results shown represent the mean of at least 3 experiments.

### Zn release from NP

10 mg of Zn-NPs (both BBB-NPs and P-NPs) loaded with ZnSO_4_ were suspended in 1 ml of milliQ water into a dialysis membrane tube (3500 MW cut-off). The dialysis membrane tube was then placed into a becker containing the release medium (25 ml of milliQ water), which was stirred continuously and maintained at 37°C. At determined intervals, 100 µl of release medium solution was collected and then replaced with 100 µl of fresh milliQ water. This sample was diluted to 25 ml with Milli-Q water and then the solution was analyzed with atomic absorption spectrophotometry. The results shown represent the mean of at least 3 experiments.

### Hippocampal culture from rat brain

The preparation of hippocampal cultures was performed essentially as described by Goslin et al. [48] with some modifications as detailed in Dresbach et al. [49]. Cell culture experiments of hippocampal primary neurons from rat (embryonic day-18; E18) were performed as described previously [50]. After preparation the hippocampal neurons were seeded on poly-l-lysine (0.1 mg/ml; Sigma-Aldrich, Steinheim) glas coverlslips. Cells were grown in Neurobasal medium (Invitrogen), complemented with B27 supplement (Invitrogen), 0.5 mM L-Glutamine (Invitrogen) and 100 U/ml penicillin/streptomycin (Invitrogen) and maintained at 37°C in 5% CO_2_. All animal experiments were performed in compliance with the guidelines for the welfare of experimental animals issued by the Federal Government of Germany and the National Institutes of Health. All of the experiments were conducted in strict compliance with APLAC approved animal protocols from Stanford University (protocol # 14607) and by the local ethics committee (Ulm University) ID Number: O.103.

### Immunhistochemistry

For immunofluorescence, HEK293 cells and primary cultures were fixed with 4% paraformaldehyde (PFA)/1.5% sucrose/PBS at 4°C for 20 min and processed for immunohistochemistry. After washing 3× 5 min with 1× PBS at RT, blocking was performed with 0.5% cold fish gelatine (Sigma) and 0.1% Ovalbumin (Sigma)/1× PBS for 30 min at RT and the cells were washed again 3× 5 min with 1× PBS at RT, followed by the primary antibody at 4°C overnight. After a 3× 5 min washing-step with 1× PBS, incubation with the second antibody coupled to Alexa488, Alexa568, Alexa647 or TexasRed (Molecular Probes) for 1 h followed. The cells were washed again in 1× PBS for 10 min and 5 min with aqua bidest and mounted in Vecta Shield mounting medium with or without DAPI (for staining the nucleus) for fluorescence microscopy. To test cell viability, the number of DAPI positive nuclei and neurons was counted from 10 optic fields for each condition.

### Zinc staining

Zn-P-NPs and Zn-BBB-NPs were suspended in Neurobasal Medium (+Glutamine +B27) and applied to cells. As Control, different solutions with defined Zn^2+^ concentration were used. An aliquot of Zinpyr-1 DMSO stock solution (5 mM) was diluted to a final concentration of 1–5 µM in culture medium and the cells were incubated with Zinpyr-1 for 20 minutes at 37°C. After this, cells were washed in Ca/Mg Phosphate buffered saline and used for confocal microscopy. Images were taken with the same exposure time and grey values quantified using ImageJ v.1.44e. For quantification, the basic fluorescence of the medium (grey value) was measured and subtracted from the grey values of a defined ZnCl_2_ solution as well as the Zn-P-NPs and Zn-BBB-NPs-suspension. The ratio of the normalized ZnCl_2_ solution to the NPs suspension determined the final Zn^2+^ concentration. Measurements were performed three times for each time point using different exposure times.

### Measurement of Zn^2+^ concentrations

The zinc-concentration of hippocampal cell culture medium was measured by Plasma-Massspectrometry (ICP-MS) at the “Spurenanalytisches Laboratorium Dr. Baumann” (Maxhütte-Haidhof, Germany).

### Statistical analysis

Images were taken with a spinning disk confocal microscope (Zeiss) using MetaMorph (Universal Imaging, Downing-town, PA) software. Quantification of fluorescence data was performed using, Image J 1.44e software with JACoP plugin. Statistical analysis in this paper was performed using Microsoft Excel for Macintosh and data were tested for significance using two-tailed, Student's t-test followed by ANOVA and p-values <0.05 were stated as significant (<0.05*; <0.01**; <0.001***).

## Supporting Information

Figure S1 **Cells treated with unloaded P-NPs and BBB-NPs exhibited no difference in their morphology and synapse density compared to untreated cells.** A) Dendrite branching was measured using MAP2 stained neurons. The dendritic complexity index (DCI) [13] was calculated based on the equation: [DCI = (# of prim. Dendrites * 1+# of sec. Dendrites * 2+# of tert. Dendrites * 3)/(# of prim.+sec.+tert. Dendrites)]. B) The number of synapses per unit length of dendrites was evaluated using Homer1 as postsynaptic marker and Bassoon as presynaptic marker. The number of Homer1/Bassoon colocalizing signals per 10 µm dendrite length is shown.
(EPS)

Figure S2 A) Overview of images of HEK293 (upper panel) and neurons (lower panel) shown in detail in Figure 2B. White squares indicate regions shown in Figure 2B. Bottom row of images in each panel are heat maps revealing regional differences in zinc levels. B) Fluorescent images of neurons 1, 2, 6 and 7 days following the addition of Rhodamine conjugated BBB-NPs. After 7 days the Rhodamine-BBB-NPs exhibit a diffuse pattern suggesting that endocytosed BBB-NPs are partially released into the cell soma.
(EPS)

Figure S3 **Prolonged exposure to high free Zn^2+^ concentration causes cell death in HEK293 cells and hippocampal neurons.** To determine the range of viable intracellular Zn^2+^ concentrations and their limit for toxicity, we supplemented cell cultures with increasing Zn^2+^ concentrations for 8 h. The results show that neurons (upper row) are more sensitive to free Zn^2+^ compared to HEK293 cells (lower row). After supplementation of the medium with a concentration of 80 µM Zn^2+^ and higher, neurons undergo cell death visible through cell fractionation, which leads to a loss of intracellular fluid and thus Zn^2+^ (full arrows). Additionally, condensed and fractionated nuclei appear (open arrow). HEK293 cells undergo cell death at Zn^2+^ concentrations higher then 160 µM, visible through cells rounding up due to a loss of surface adhesion.
(EPS)

Figure S4 **Supplementation of HEK293 cells (left panel) and hippocampal neurons (right panel) with ZnCl_2_ was used as reference for the evaluation of zinc concentrations (** **Fig. 4** **).** The brightness of the Zinpyr-1 signal of untreated cells and cells treated with a defined amount (30 µM) ZnCl_2_ was used to evaluate the increase in zinc concentration by Zn-P-NPs and Zn-BBB-NPs (Fig. 4). The final zinc concentration was calculated using grey values of Zinpyr-1 fluorescence. Background fluorescence of untreated neurons was measured and cells treated with 30 µM ZnCl_2_ used as reference. HEK293 cells were supplemented with ZnCl_2_ at DIV0 and the zinc concentration was evaluated after 1 and 3 days. Hippocampal neurons were supplemented with ZnCl_2_ at DIV6 and the zinc concentration was evaluated at DIV7 and DIV14.
(EPS)

## References

[1] Pardridge WM (2003). Blood Brain Barrier drug targeting: the future of brain drug
development.. Mol Interv.

[2] Burke M, Langer R, Brim H (1999). Central Nervous System: Drug Delivery to Treat.

[3] Costantino L, Gandolfi F, Tosi G, Rivasi F, Vandelli MA (2005). Peptide-derivatized biodegradable nanoparticles able to cross the
blood–brain barrier.. Journal of Controlled Release.

[4] Tosi G, Costantino L, Rivasi F, Ruozi B, Leo E (2007). Targeting the central nervous system: in vivo experiments with
peptide-derivatized nanoparticles loaded with Loperamide and
Rhodamine-123.. J Control Rel.

[5] Tosi G, Costantino L, Rivasi F, Ruozi B, Leo E (2007). Targeting the central nervous system: in vivo experiments with
peptide-derivatized nanoparticles loaded with Loperamide and
Rhodamine-123.. J Control Release.

[6] Tosi G, Vergoni AV, Ruozi B, Bondioli L, Badiali L (2010). Sialic acid and glycopeptides conjugated PLGA nanoparticles for
central nervous system targeting: In vivo pharmacological evidence and
biodistribution.. J Control Release.

[7] Vergoni AV, Tosi G, Tacchi R, Vandelli MA, Bertolini A (2009). Nanoparticles as drug delivery agents specific for CNS: in vivo
biodistribution.. Nanomedicine.

[8] Levenson CW (2006). Zinc: The New Antidepressant?. Nutrition Reviews.

[9] Cope EC, Levenson CW (2010). Role of zinc in the development and treatment of mood
disorders.. Curr Opin Clin Nutr Metab Care.

[10] Tassabehji NM, Corniola RS, Alshingiti A, Levenson CW (2008). Zinc deficiency induces depression-like symptoms in adult
rats.. Physiol Behav.

[11] Nowak G, Szewczyk B, Pilc A (2005). Zinc and depression. An update.. Pharmacol Rep.

[12] Whittle N, Lubec G, Singewald N (2009). Zinc deficiency induces enhanced depression-like behaviour and
altered limbic activation reversed by antidepressant treatment in
mice.. Amino Acids.

[13] Lom B, Cohen-Cory S (1999). Brain-derived neurotrophic factor differentially regulates
retinal ganglion cell dendritic and axonal arborization in
vivo.. J Neurosci.

[14] Hansen GH, Rasmussen K, Niels-Christiansen LL, Danielsen EM (2009). Endocytic trafficking from the small intestinal brush border
probed with FM dye.. Am J Physiol Gastrointest Liver Physiol.

[15] Yokoyama M, Koh J, Choi DW (1986). Brief exposure to zinc is toxic to cortical
neurons.. Neurosci Lett.

[16] Weiss JH, Hartley DM, Koh JY, Choi DW (1993). AMPA receptor activation potentiates zinc
neurotoxicity.. Neuron.

[17] Canzoniero LM, Turetsky DM, Choi DW (1999). Measurement of intracellular free zinc concentrations
accompanying zinc-induced neuronal death.. J Neurosci.

[18] Tosi G, Costantino L, Ruozi B, Forni F, Vandelli MA (2008). Polymeric nanoparticles for the drug delivery to the Central
Nervous System.. Exp Opin Drug Del.

[19] Shi N, Pardridge WM (2000). Noninvasive gene targeting to the brain.. Prot Natl Acad Sci USA.

[20] Vergoni AV, Tosi G, Tacchi R, Vandelli MA, Bertolini A (2009). Nanoparticles as drug delivery agents specific for CNS: in vivo
biodistribution.. Nanomedicine: Nanotechnology, Biology and Medicine.

[21] Garcia-Garcia E, Andrieux K, Gil S, Couvreur P (2007). Colloidal carriers and blood-brain barrier (BBB) translocation: a
way to deliver drugs to the brain?. Int J Pharm.

[22] Kreuter J (2007). Nanoparticles- a historical perspective.. Int J Pharm.

[23] Qiao F, Bowie JU (2005). The many faces of SAM.. Sci STKE.

[24] Baron MK, Böckers TM, Vaida B, Faham S, Gingery M (2006). An Architectural Framework That May Lie at the Core of the
Postsynaptic Density.. Science.

[25] Gundelfinger ED, Boeckers TM, Baron MK, Bowie JU (2006). A role for zinc in postsynaptic density asSAMbly and
plasticity?. Trends Biochem Sci.

[26] Grabrucker AM, Knight MJ, Proepper C, Bockmann J, Joubert M (2011). Concerted action of zinc and ProSAP/Shank in synaptogenesis and
synapse maturation.. Embo Journal.

[27] Kroczka B, Brañski P, Palucha A, Pilc A, Nowak G (2001). Antidepressant-like properties of zinc in rodent forced swim
test.. Brain Res Bull.

[28] Kroczka B, Ziêba A, Dudek D, Pilc A, Nowak G (2002). Zinc exhibits an antidepressant-like effect in the forced
swimming test in mice.. Pol J Pharmacol.

[29] Nowak G, Szewczyk B, Wieroñska JM, Brañski P, Palucha A (2003). Antidepressant-like effects of acute and chronic treatment with
zinc in forced swim test and olfactory bulbectomy model in
rats.. Brain Res Bull.

[30] Rosa AO, Lin J, Calixto JB, Santos AR, Rodrigues AL (2003). Involvement of NMDA receptors and L-arginine-nitric oxide pathway
in the antidepressant-like effects of zinc in mice.. Behav Brain Res.

[31] Cunha MP, Machado DG, Bettio LE, Capra JC, Rodrigues AL (2008). Interaction of zinc with antidepressants in the tail suspension
test.. Prog Neuropsychopharmacol Biol Psychiatry.

[32] Szewczyk B, Brañski P, Wieroñska JM, Palucha A, Pilc A (2002). Interaction of zinc with antidepressants in the mouse forced
swimming test.. Pol J Pharmacol.

[33] Manser WWT, Khan MA, Hasan KZ (1989). Trace element studies on Karachi population. Part IV: blood
copper, zinc, magnesium and lead levels in psychiatric patients with
depression, mental retardation and seisure disorders.. J Pakistan Med Assoc.

[34] McLoughlin IJ, Hodge SJ (1990). Zinc in depressive disorder.. Acta Psychiatr Scand.

[35] Nowak G, Szewczyk B (2002). Mechanism contributing to anti-depressant zinc
actions.. Pol J Pharmacol.

[36] Maes M, D'Haese PC, Scharpe S, D'Hondt PD, Cosyns P (1994). Hypozincemia in depression.. J Affect Disord.

[37] Wójcik J, Dudek D, Schlegel-Zawadzka M, Grabowska M, Marcinek A (2006). : Antepartum/postpartum depressive symptoms and serum zinc and
magnesium levels.. Pharmacol Rep.

[38] Sawada T, Yokoi K (2010). Effect of zinc supplementation on mood states in young women: a
pilot study.. Eur J Clin Nutr.

[39] Amani R, Saeidi S, Nazari Z, Nematpour S (2010). Correlation between dietary zinc intakes and its serum levels
with depression scales in young female students.. Biol Trace Elem Res.

[40] Tosi G, Fano RA, Bondioli L, Badiali L, Benassi R (2010). Investigation on Mechanisms of Glycopeptide Nanoparticles for
Drug Delivery across the Blood-Brain Barrier,. Nanomedicine.

[41] Huttner WB, Dotti CG (1991). Exocytotic and endocytotic membrane traffic in
neurons.. Curr Opinion in Neurobiology.

[42] Depino AM, Earl C, Kaczmarczyk E, Ferrari C, Besedovsky H (2003). Microglial activation with atypical proinflammatory cytokine
expression in a rat model of Parkinson's disease.. Eur J Neurosci.

[43] Marchetti B, Serra PA, L'Episcopo F, Tirolo C, Caniglia S (2005). Hormones are key actors in gene×environment interactions
programming the vulnerability to Parkinson's disease: glia as a common
final pathway.. Ann NY Acad Sci.

[44] Mattson MP, Chan SL (2003). Neuronal and glial calcium signaling in Alzheimer's
disease.. Cell Calcium.

[45] Schubert P, Ferroni S, Aschner Michael, Costa LucioG. (2005). Role of microglia and astrocytes in Alzheimer's
disease,. The Role of Glia in Neurotoxicity, second ed..

[46] Blanco MD, Alonso MJ (1997). Development and characterization of protein-loaded
poly(lactide-co-glycolide) nanospheres.. Eur J Pharm Biopharm.

[47] Liu Y, Li K, Liu B, Feng S-S (2010). A strategy for precision engineering of nanoparticles of
biodegradable copolymers for quantitative control of targeted drug
delivery,. Biomaterials.

[48] Goslin K, Banker G (1991). Rat hippocampal neurons in low density culture, in Culturing Nerve
Cells.

[49] Dresbach T, Hempelmann A, Spilker C, tom Dieck S, Altrock WD (2003). Functional regions of the presynaptic cytomatrix protein bassoon:
significance for synaptic targeting and cytomatrix
anchoring.. Cell Neurosci.

[50] Seidenbecher CI, Langnaese K, Sanmarti-Vila L, Boeckers TM, Smalla KH (1998). Caldendrin, a novel neuronal calcium-binding protein confined to
the somato-dendritic compartment.. J Biol Chem.

